# A new cryptic species of *Echinostoma* (Trematoda: Echinostomatidae) closely related to *Echinostoma paraensei* found in Brazil

**DOI:** 10.1017/S003118202300001X

**Published:** 2023-04

**Authors:** Marisa C. Valadão, Philippe V. Alves, Danimar López-Hernández, Jordana C. A. Assis, Paulo R. S. Coelho, Stefan M. Geiger, Hudson A. Pinto

**Affiliations:** 1 Department of Parasitology, Institute of Biological Sciences, Universidade Federal de Minas Gerais, P.O. Box 486, 30123-970, Belo Horizonte, Minas Gerais, Brazil; 2 Section of Parasitology, Institute of Biosciences, São Paulo State University (UNESP), 18618-689, Botucatu, São Paulo, Brazil

**Keywords:** Brazil, cryptic species, *Echinostoma*, integrative taxonomy, phylogeny

## Abstract

*Echinostoma paraensei*, described in Brazil at the end of the 1960s and used as a biological model for a range of studies, belongs to the ‘*revolutum*’ complex of *Echinostoma* comprising species with 37 collar spines. However, molecular data are available only for a few isolates maintained under laboratory conditions, with molecular prospecting based on specimens originating from naturally infected hosts virtually lacking. The present study describes *Echinostoma maldonadoi* Valadão, Alves & Pinto n. sp., a species cryptically related to *E. paraensei* found in Brazil. Larval stages (cercariae, metacercariae and rediae) of the new species were found in the physid snail *Stenophysa marmorata* in the State of Minas Gerais, Brazil, the same geographical area where *E. paraensei* was originally described. Adult parasites obtained experimentally in *Meriones unguiculatus* were used for morphological (optical microscopy) and molecular [28S, internal transcribed spacer (ITS), *nad*1 and *cox*1] characterization. The morphology of larval and adult parasites (most notable the small-sized dorsal spines in the head collar), associated with low (0–0.1%) molecular divergence for 28S gene or ITS region, and only moderate divergence for the mitochondrial *cox*1 gene (3.83%), might suggest that the newly collected specimens should be assigned to *E. paraensei*. However, higher genetic divergence (6.16–6.39%) was found in the mitochondrial *nad*1, revealing that it is a genetically distinct, cryptic lineage. In the most informative phylogenetic reconstruction, based on *nad*1, *E. maldonadoi* n. sp. exhibited a strongly supported sister relationship with *E*. *paraensei*, which may indicate a very recent speciation event giving rise to these 2 species.

## Graphical abstract

**Figure:**
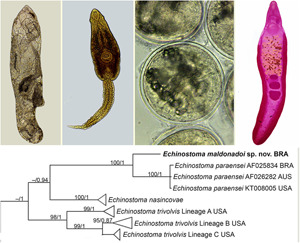

## Introduction

Species of the genus *Echinostoma* Rudolphi, 1809 are digenetic trematodes found, in the adult stage, in the intestine of birds and mammals, including humans (Fried and Graczyk, 2000; Toledo *et al*., 2009; Chai *et al*., 2020). More than 120 nominal species have been described worldwide making it the most diverse genus in the Echinostomatidae (Kostadinova and Gibson, 2000), but the pronounced morphological uniformity among species, along with other historical issues, has led to a complex taxonomic scenario. This is particularly true for the ‘*revolutum*’ group, formed by species of *Echinostoma* possessing 37 collar spines. About 60 nominal species were described worldwide (Chai *et al*., 2020), but the validity of most of them still requires confirmation, given that this group has been subjected to intense debate and constant taxonomic rearrangements over time (Georgieva *et al*., 2013, 2014; Faltýnková *et al*., 2015).

In the last decades, the use of molecular tools has revealed the existence of numerous independent evolutionary lineages of *Echinostoma*, usually referred to as cryptic species, but also has helped to unravel the correct identity of previously known taxa, some of them described as new (Detwiler *et al*., 2010; Georgieva *et al*., 2013, 2014, 2017; Faltýnková *et al*., 2015; Izrailskaia *et al*., 2021; Valadão *et al*., 2022). These recent advances have contributed to a better knowledge of the species composition and the distribution of these parasites and have generated evidence on their biogeography, evolution and radiation. However, such integrative taxonomic studies involving *Echinostoma* spp. are disproportionately carried out in Asia, Europe and North America (Detwiler *et al*., 2010; Georgieva *et al*., 2014; Chai *et al*., 2021). In fact, in South America, molecular studies involving species of *Echinostoma* are scarce (Maldonado *et al*., 2001*a*; Valadão *et al*., 2022) and do not reflect the diversity of species known in this subcontinent (more than 30 species, 12 bearing 37 collar spines) (Travassos *et al*., 1969; Fernandes *et al*., 2015; Lunaschi *et al*., 2018; Valadão *et al*., 2022).

Among the species belonging to the ‘*revolutum*’ group is *Echinostoma paraensei* Lie and Basch, 1967, described based on adult parasites recovered in the course of experimental studies initiated from naturally infected *Biomphalaria glabrata* (Say, 1818) collected in Brazil. The main differential trait of the species is the relatively small size of the dorsal collar spines (Lie and Basch, 1967). Some decades later, the semi-aquatic rodent, *Nectomys squamipes* (Brants, 1827), was identified as a natural definitive host in Brazil (Maldonado *et al*., 2001*a*). The life cycle of the species is maintained under laboratory conditions in the USA and Brazil (Toledo and Fried, 2005; Fried and Peoples, 2009), which made it possible to carry out several biological studies involving *E. paraensei*. These studies contributed to the knowledge of several aspects related to this species, including (i) parasite behaviour (e.g. Loker *et al*., 1987; Nollen, 1996; Maldonado *et al*., 2005), (ii) parasite interaction (in both intramolluscan and vertebrate phases) with *Schistosoma mansoni* Sambon, 1907 and/or other echinostomes (e.g. Sullivan and Richards, 1981; Combes, 1982; Maldonado *et al*., 2001*b*; Garcia *et al*., 2010; Hanington *et al*., 2010), (iii) ultrastructural morphology of different developmental stages (e.g. Fujino *et al*., 2000; Maldonado *et al*., 2001*a*; Pinheiro *et al*., 2005; Souza *et al*., 2017*a*), (iv) biochemical and pathological changes associated with the infection in molluscs and rodents (e.g. Adema *et al*., 2000; Toledo, 2009; Garcia *et al*., 2011, 2021; Souza *et al*., 2017*a*), (v) parasite protein composition (proteomics) (e.g. Loker *et al*., 1992; Marcilla, 2009) and (vi) sequencing of expressed sequence tags (Adema *et al*., 2000; Nowak and Loker, 2005).

Despite this tremendous scientific knowledge accumulated over decades about *E. paraensei*, studies on the molecular characterization of the species are scarce. Considering genetic markers, commonly used in taxonomic studies of trematodes, few sequences generated for *E. paraensei* are available in the public genetic sequence databases. They correspond to the complete mitochondrial genome of the isolate maintained under laboratory conditions in the USA (KT008005 – Adema *et al*., direct submission in GenBank^®^, unpublished), some internal transcribed spacer (ITS) sequences generated for the Brazilian isolate from *N. squamipes* (Maldonado *et al*., 2001*a*) and *nad*1 sequences obtained for larval stages found in Australia and for the isolate originated from Brazil and maintained in the USA (Morgan and Blair, 1998*a*).

In the present study, we provide data obtained during experimental, morphological and molecular studies of a 37-collar-spined echinostome found in *Stenophysa marmorata* (Guilding, 1828) from Brazil. Despite the morphological data of the developmental stages and sequences of nuclear genetic markers (28S and ITS) suggested that the parasite is indistinguishable from *E. paraensei*, mitochondrial data (*nad*1 gene) pointed to a genetically distinct lineage, herein described as a new species of *Echinostoma*.

## Materials and methods

### Sampling and experimental infection

The isolate of the parasite evaluated in this study was obtained from a specimen of *S. marmorata* collected in the municipality of Alvorada de Minas, Minas Gerais, Brazil, in February 2019 during a malacological survey focused on the epidemiology of schistosomiasis. Details of malacological techniques and preliminary evaluation of infection with larval trematodes are as described in Coelho *et al*. (2021, 2022). The infected snail was sent to the laboratory for further parasite identification. A new photostimulation test was carried out for the recovery of cercariae. The infected mollusc died on the following day and was dissected for retrieval of rediae and metacercariae. This fact precludes us from providing detailed illustrations of larval stages.

For the recovery of adult parasites, part of the collected metacercariae were used to infect a laboratory-reared jird, *Meriones unguiculatus* (Milne-Edwards, 1867). The rodent was orally inoculated with approximately 60 metacercariae. The infected animal was submitted to an immunosuppression protocol through subcutaneous inoculation of dexamethasone disodium phosphate (Decadron, Ache, Brazil), 25 mg kg^−1^, daily until the end of the experiment. The success of infection was verified by fecal examination through the spontaneous sedimentation method (Lutz, 1919) from 7 days post-infection (dpi) onwards. The animal was kept in the laboratory with water and food provided *ad libitum* until the euthanasia carried out at 15 dpi. The intestine was transferred to Petri dishes containing physiological saline (0.85% NaCl) and examined under a stereomicroscope for the presence of adult or juvenile parasites.

### Morphological study

Samples of the emerged cercariae were stained with vital stains (0.05% neutral red or Nile blue sulphate) and mounted in temporary preparations for examination under a light microscope. Larvae were also killed in hot water and fixed in 10% formalin for morphometric analysis. Rediae and metacercariae were studied live in temporary preparations and then also fixed in 10% formalin. Adult parasites collected from the experimentally infected jird were individually compressed between glass slides and fixed in 10% formalin. Later, they were stained with alum acetocarmine, dehydrated in an increasing series of ethanol, clarified in beechwood creosote and mounted on permanent slides with Canada balsam. For the study of the cephalic collar, some specimens had their anterior extremity sectioned with the aid of a scalpel blade, transferred to a glass slide and clarified with Amman's lactophenol. The preparations were examined under the microscope for determination of the number, size, shape and arrangement of collar spines, according to Fried *et al*. (2009). Whole adult parasites were photographed using a digital camera Samsung^®^ ES70 (Samsung, Brazil), coupled to a stereomicroscope. Details of structures of adults and the larval stages were photographed with a Leica ICC50 HD digital camera, coupled to a Leica DM500 microscope. Captured images were analysed with Leica Application Suite software (LAZ EZ), version 2.0 (Leica Microsystems, Germany). The drawings were made with the aid of a camera lucida connected to an Olympus BH-2 microscope (Olympus, Japan). The morphometric analyses were carried out using a micrometre eyepiece. The abbreviations used in the morphological descriptions are in accordance with the most recent descriptions of *Echinostoma* (Kostadinova, 2005, Faltýnková *et al*., 2015; Georgieva *et al*., 2017). In the case of dorsal spines (DLSL, length of the dorsolateral spines; DMSL, length of the most dorsal spines), we used the terminology presented by Maldonado *et al*. (2001*a*). Data obtained were compared with descriptions available to *Echinostoma* spp. reported in South America (Lie and Basch, 1967; Travassos *et al*., 1969; Maldonado *et al*., 2001*a*, 2003; Fernandes *et al*., 2015).

The type material is deposited in the Helminthological Collection of the Institute Oswaldo Cruz (CHIOC) and Collection of Trematodes of the Federal University of Minas Gerais (UFMG-TRE).

### Molecular study

An adult specimen fixed in 95% ethanol was used for DNA extraction using the QIAamp^®^ DNA Mini kit (Qiagen, USA), according to manufactures' instructions. The concentration and quality of extracted DNA was evaluated using the microvolume spectrophotometer NanoDrop^®^ Lite (Thermo Fisher Scientific, Rockford, IL, USA). Partial regions of 28S rRNA gene (primers Dig-12/1500R; Tkach *et al*., 2003), ITS1-5.8S-ITS2 regions (primers BD1/BD2; Luton *et al*., 1992), *cox*1 gene (JB3/CO1-R trema; Miura *et al*., 2005) and *nad*1 gene (primers NDJ11/NDJ2a; Kostadinova *et al*., 2003) were amplified by PCR. The amplification reactions were carried out in a final volume of 25 *μ*L, including 12.5 *μ*,L of Platinum Hot Start Master Mix 2 × (Invitrogen, Thermo Fisher Scientific Baltics, Vilnius, Lithuania), 1.25 pmol of each primer, about 50 ng of template DNA and sterile ddH_2_O. PCR products were electrophoresed on a 1.5% agarose gel, and the band of the expected size was purified with 20% polyethylene glycol 8000 (Promega, Madison, WI, USA). Purified DNA was sequenced in both directions by capillary electrophoresis in an ABI3730 sequencer using the Big Dye Terminator Cycle Sequencing kit (Applied Biosystems, Foster City, CA, USA). The chromatograms obtained were assembled using ChromasPro v.2.0.1 software (Technelysium Pty Ltd, South Brisbane, Queensland, Australia) and the generated contiguous sequences used in the subsequent analyses.

The newly generated sequences were first subjected to a search in the Basic Local Alignment Search Tool – BLAST (Altschul *et al*., 1990) (https://blast.ncbi.nlm.nih.gov) in GenBank. Data obtained for the 4 molecular markers evaluated were compared exclusively with congeneric species. Alignment constructions were performed using the algorithm MUSCLE implemented in the software MEGA X (Kumar *et al*., 2018). Before phylogenetic analyses, alignments were trimmed to match the shortest sequence. The evolutionary models used for the analyses were estimated by the Bayesian information criterion implemented in MEGA X. Outgroups for each dataset were chosen based on previous molecular phylogenetic studies including representatives of *Echinostoma* and related taxa (Sereno-Uribe *et al*., 2015; Tkach *et al*., 2016; Georgieva *et al*., 2017; Nakao and Sasaki, 2021). The methods used to generate the phylogenetic hypotheses were maximum likelihood (ML) and Bayesian inference (IB). ML analyses were performed in MEGA X and nodal support was measured using the bootstrap test with 1000 replicates. BI analyses were performed with MrBayes v.3.2.6 software (Ronquist *et al*., 2012) and posterior probability values were determined by the Monte Carlo method *via* Markov chains, in 2 simultaneous runs of 4 chains for 10^6^ generations, with topologies sampling every 100 generations. The ‘burn-in’ was established for the first 25% of the trees sampled, and the nodal support was estimated from the remaining trees. The trees obtained by BI were visualized with FigTree software version 1.4.3 (http://tree.bio.ed.ac.uk/software/figtree/) and edited in Adobe Photoshop^®^ v.22.4.3 software (Adobe Inc., USA). All sequences generated in this study were deposited in GenBank under the accession numbers OQ132569 (28S), OQ132538 (ITS1-5.8S-ITS2), OQ134393 (*cox*1) and OQ126877 (*nad*1).

## Results

A total of 56 specimens of a 37-collar-spined *Echinostoma* were recovered from the small intestine of *M. unguiculatus* 15 days after the experimental infection with metacercariae, obtained from *S. marmorata*. The obtained morphological and molecular data pointed to a new species of *Echinostoma* from the ‘*revolutum*’ group, which is described below.

Family Echinostomatidae Looss, 1899

Genus *Echinostoma* Rudolphi, 1809

### *Echinostoma maldonadoi* Valadão, Alves & Pinto n. sp.

Natural intermediate host: *Stenophysa marmorata* (Guilding, 1828) (Gastropoda: Physidae)

Prevalence of infection: 1/224 (0.45%).

Experimental definitive host: *Meriones unguiculatus* (Milne-Edwards, 1867) (Rodentia: Muridae).

Natural definitive host: unknown.

Type locality: Alvorada de Minas (18°43′7″ S 43°22′5″ W), Minas Gerais, Brazil

Type material: Holotype (CHIOC 39915 a); paratypes (5 specimens, CHIOC 39915 b-f and 10 specimens, UFMG–TRE UFMG-TRE 133).

Representative DNA sequences: 28S (OQ132569), ITS1-5.8S-ITS2 (OQ132538), *cox*1 (OQ134393) and *nad*1 (OQ126877).

ZooBank registration: urn:lsid:zoobank.org:pub:2A535C25-8E19-4716-8E6F-B94AC683A7C6

Etymology: The specific name *maldonadoi* is given to honour the Brazilian helminthologist, Dr. Arnaldo Maldonado Júnior, for his relevant contributions to the biology and taxonomy of *Echinostoma*.

#### Description

**Fig. 1.**
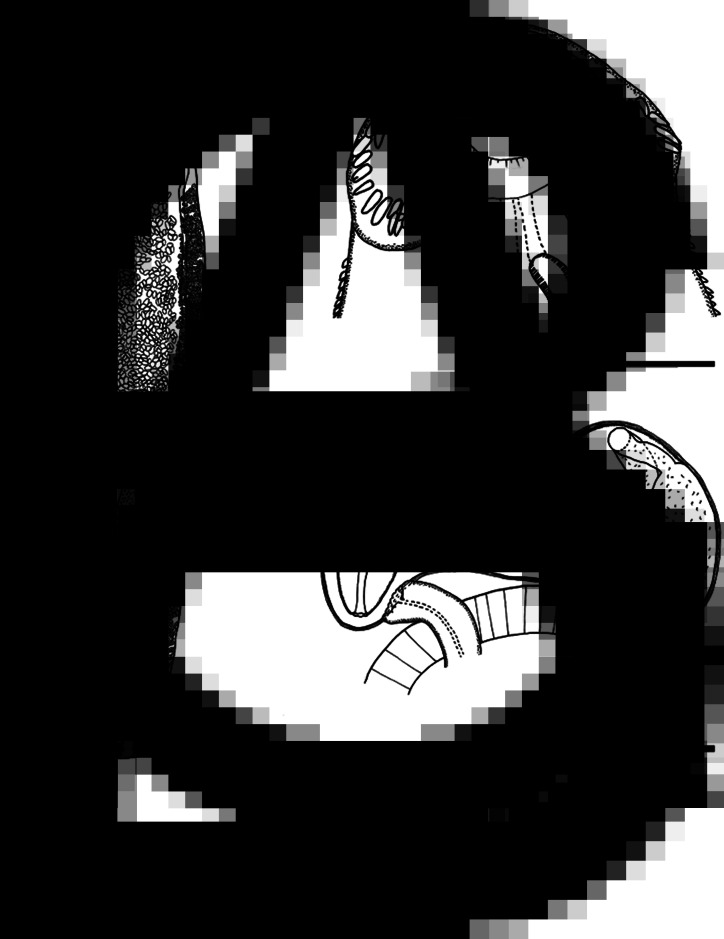
*Echinostoma maldonadoi* n. sp. line drawings of the holotype. (A) Whole view. (B) Detail of cephalic collar. (C) Detail of genital complex. Scale bars: A: 1 mm; B, C: 250 *μ*m.

**Fig. 2.**
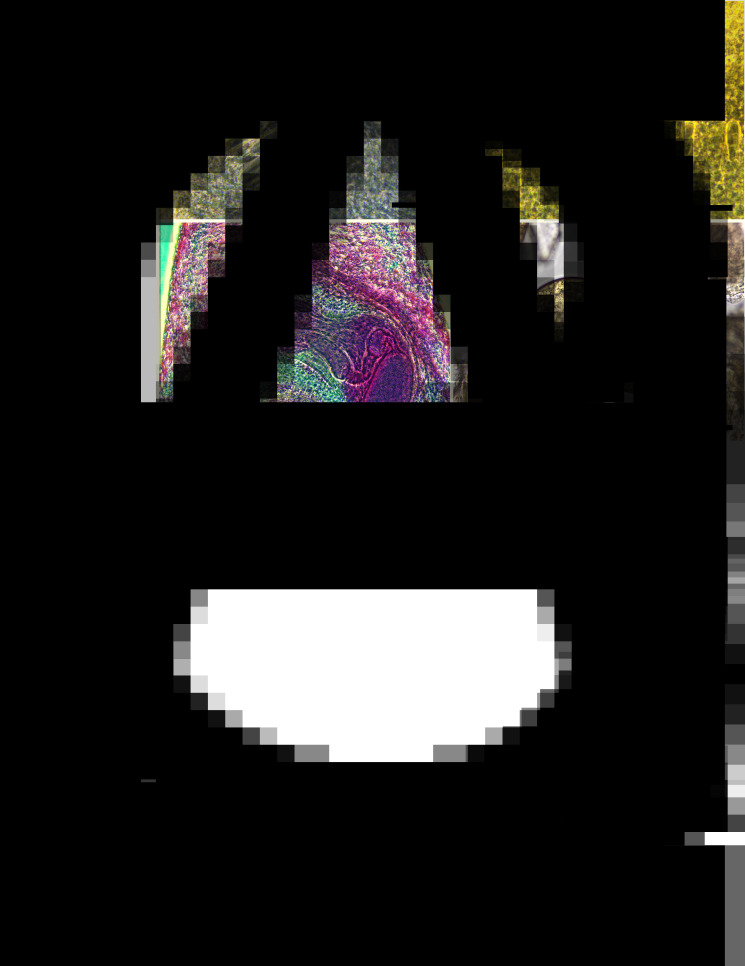
Micrographs of *Echinostoma maldonadoi* n. sp. (A) Whole view of a carmine-stained paratype. (B) Detail of angular (*) and lateral (#) spines. (C) Detail of dorsal spines (+). (D) Detail of the cirrus sac. (E) Eggs obtained in feces. Scale bars: A: 1 mm; B, C, E: 50 *μ*m; D: 100 *μ*m.

*Adult* (*n* = 24, Figs 1 and 2; measurements in Table 1): Body slender, elongate, dorsoventrally flattened, with maximum width at uterine level, 4.5–6.4 times longer than wide, with almost parallel lateral margins and notable constriction at posterior margin of ventral sucker. Tegument with scale-like spines in alternating transverse rows, denser at pharynx region, extending to level of posterior margin of ventral sucker and reaching posterior testis level on ventral surface. Forebody short, 9–15% of body length. Head collar (*n* = 10) well developed, reniform, width representing 18–34% of maximum body width. Collar spines conspicuous, 37 in number in most specimens (5 with 37, 2 with 33, 1 with 36, 38 or 39 spines), with following arrangement: 5 angle spines (3 oral and 2 aboral); 6 lateral spines in single row on each ventral lappet, and 15 dorsal spines in double row (8 oral and 7 aboral). Dorsal spines varying in length, with the dorsolateral spines markedly larger than central (dorsalmost) spines. Oral sucker ventro-subterminal, rounded, muscular. Prepharynx very short; pharynx oval to elongate-oval, muscular. Oesophagus short, 2–4% of body length; oesophageal bifurcation just anterior to ventral sucker; intestinal caeca blind, almost reaching posterior extremity. Ventral sucker spherical, muscular, located in the first quarter of body; 2 times as large as oral sucker (suckers–width ratio 1:2.1–2.4). Testes 2, subglobular, smooth or slightly indented, in tandem, located in third quarter of body; post-testicular region (between posterior testis and posterior extremity) corresponding to 25–44% of body length. Cirrus sac elongate-oval, anterior to ventral sucker, with a simple elongate-oval seminal vesicle, well-developed pars prostatica, coiled ejaculatory duct and unspined cirrus. Genital pore at the level of anterior margin of ventral sucker. Ovary transversely oval, post-equatorial, pretesticular. Mehlis' gland contiguous with ovary. Uterus intercaecal, with numerous coils between ventral sucker and ovary; uterine field corresponding to 24–32% of body length. Metraterm short, weakly muscular. Vitellarium follicular, forming 2 lateral non-confluent fields of small follicles overlapping caeca and extending from level of the region slightly posterior to ventral sucker to posterior extremity of body. Eggs abundant, oval, yellow, operculated, immature.

**Table 1.** Morphometric data for adults of *Echinostoma maldonadoi* n. sp. and isolates of *Echinostoma paraensei* and *Echinostoma pseudorobustum*

	*Echinostoma maldonadoi* n. sp.	*Echinostoma paraensei*			*Echinostoma pseudorobustum*
Hosts	*Stenophysa marmorata*^N^; *Meriones unguiculatus*^E^	*Biomphalaria glabrata*^N,E^; *Physella acuta*^E^; hamsters^E^, mice^E^, rats^E^	*B. glabrata*^E^; hamsters^E^	*Nectomys squamipes*^N^, *B. glabrata*^E^, hamsters^E^	*Gallus domesticus*^N^
Specimens and condition	*n* = 24, 15 dpi	*n* = 30, 12–109 dpi	BH Isolate, *n* = 6, 28 dpi	RJ Isolate, *n* = 30, 28 dpi	*n* = 2
References	This study	Lie and Basch (1967)	Maldonado *et al*. (2001*a*)		Valadão *et al*. (2022)
BL (mm)	10.8 ± 0.7 (9.1–12.2)	7.5–16.0	16.3 ± 159	15.5 ± 1.4	10.9–11.5
BW (mm)	2.0 ± 0.2 (1.8–2.4)	0.8–2.0	2.4 ± 80	2.4 ± 157	1.6–1.8
BW (%)	19 (15–22)	11.9*	–	13.4*	14.3–16.1
FORE (mm)	1.3 ± 0.2 (1.0–1.6)	1.6*	–	2.9*	2.1
FORE (%)	12 (9–16)	10.9*	–	13.7*	18.5–19.3
CL	291 ± 22 (250–339)	408–828	–	392*	255–309
CW	539 ± 43 (37–588)	276–660	545 ± 79	657 ± 96	801–855
CW/BW	27 (18–34)	37.4*	–	24.9*	45.8–53.2
Spines	37 (33–39)	37	36	35–37	37
AOSL	48 ± 6 (35–64)	36–99	77.7 ± 5.5	44.2 ± 5.8	39–77
AASL	48 ± 6 (31–60)	36–99	79.4 ± 9.4	45.5 ± 5.6	39–77
LSL	55 ± 6 (42–68)	36–99	74.1 ± 13.6	52.2 ± 5.1	36–107
DLSL	52 ± 6 (38–64)	22–33	56.9 ± 6.9	38.0 ± 8.1	30–84
DMSL	32 ± 8 (21–56)	22–33	–	37.5 ± 7.6	30–84
OL	330 ± 42 (273–400)	360–664	388 ± 54	405 ± 73	1.1
OL (%)	3 (2–4)	3.56*	–	1.54*	9.8–10
OSL	206 ± 18 (178–232)	347*	255 ± 28	338 ± 50	200–255
OSW	312 ± 24 (273–364)	216–408	274 ± 33	384 ± 60	309–346
VSL	780 ± 34 (728–837)	695*	870 ± 38	824 ± 65	746–765
VSW	765 ± 21 (728–801)	456–1008	917 ± 75	930 ± 108	801–837
OSW/VSW	1:2.5 (1:2.1–2.4)	1:2.8	–	1:1.4	1:2.3–2.7
PL	41 ± 12 (18–53)	72–120	139 ± 26	129 ± 42	87–123
PHL	289 ± 32 (182–346)	180–348	198 ± 26	287 ± 57	182
PHW	190 ± 20 (160–214)	180–288	161 ± 16	231 ± 42	182
ATL	597 ± 118 (255–765)	384–732	752 ± 179	844 ± 132	528–710
ATW	740 ± 78 (491–910)	312–612	717 ± 108	763 ± 95	492–583
PTL	772 ± 91 (491–892)	408–852	917 ± 99	940 ± 110	564–765
PTW	726 ± 58 (582–856)	312–516	741 ± 113	750 ± 83	473–710
TEND (mm)	4 ± 1 (3–5)	5.39*	–	5.7*	2.0–2.3
TEND (%)	34 (25–44)	35.7*	–	27.1*	18.4–19.8
OVL	384 ± 32 (328–437)	279*	490 ± 39	476 ± 86	273–364
OVW	556 ± 59 (400–655)	250–408	682 ± 51	712 ± 54	328–492
OVAR (mm)	3 ± 1 (2–4)	4.8*	–	7.2*	4.5–4.8
OVAR (%)	28 (24–32)	31.8*	–	34.4*	38.8–44.4
Eggs (L)	112 ± 4 (105–120)	104–122	–	120 ± 5	92–135
Eggs (W)	79 ± 3 (72–84)	74–86	–	85 ± 4	42–71

^N^ = natural hosts; ^E^ = experimental host; * = estimated from the published drawing; – = data unavailable.

Measurements are in micrometres, unless otherwise stated.

#### Larval stages

**Fig. 3.**
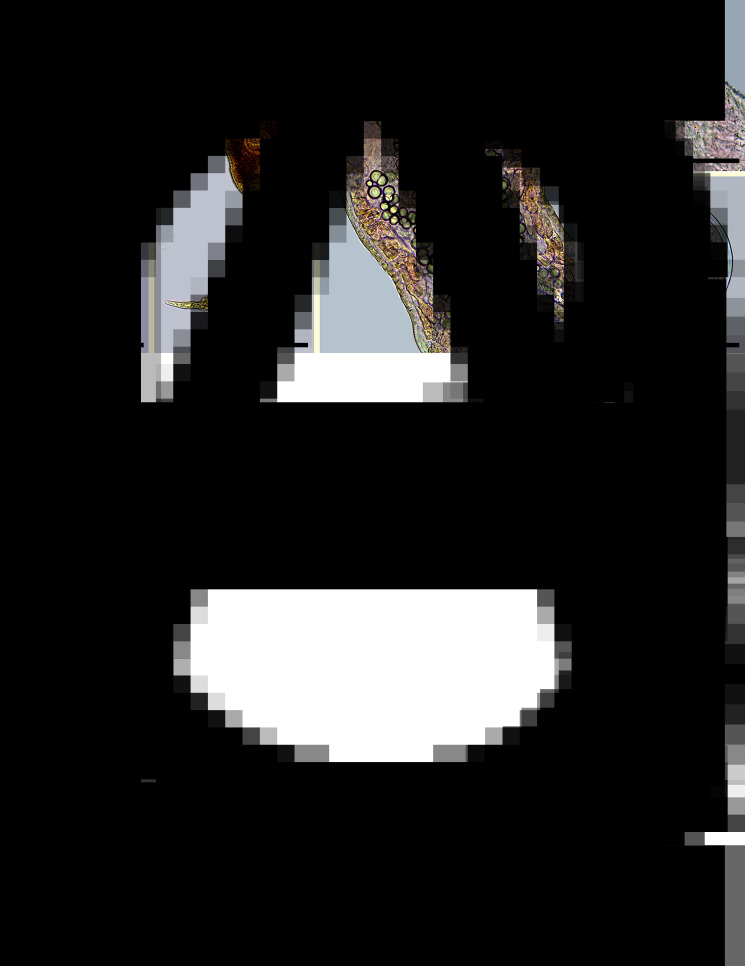
*Echinostoma maldonadoi* n. sp. larval stages found in *Stenophysa marmorata* from Brazil. (A) Redia. (B) Cercaria. (C) Detail of the body of cercaria. (D) Detail of anterior end. (E) Metacercaria. Scale bars: A: 200 *μ*m; B: 100 *μ*m; C, E: 50 *μ*m; D: 20 *μ*m.

*Mature redia* (*n* = 23, Fig. 3A; Table 2): Body elongate, tapered at its anterior and posterior ends, with yellowish pigment. Collar absent. Pharynx subspherical, muscular. Caecum short, with dark pigment inside. Birth pore prominent at the anterior region, lateral. Two prominent locomotor appendages present at about 2/3 of body length.

**Table 2.** Morphometric data of larval developmental stages of *Echinostoma maldonadoi* n. sp. and *Echinostoma paraensei* from Brazil

		*Echinostoma maldonadoi* n. sp.	*Echinostoma paraensei*
Hosts		*Stenophysa marmorata*^N^	*Biomphalaria glabrata*^NE^; *B. straminea*^E^; *Physella acuta*^E^
References		This study	Lie and Basch (1967)
*Cercaria*			
Body	L	267 ± 20 (218–310)	228–275
	W	92 ± 10 (73–109)	117–136
Oral sucker	L	37 ± 4 (28–50)	41.7*
	W	36 ± 2. (28–42)	35–40
Pharynx	L	19 ± 2 (18–27)	16–25
	W	18 ± 1 (16–18)	17–22
Ventral sucker	L	49 ± 6 (35–57)	41–51
	W	50 ± 5 (35–57)	41–51
Oral sucker/ventral sucker		1.4 (1:1.0–1.6)	1:1.4*
Tail	L	397 ± 37 (345–455)	412–490
	W	36	41–47
Tail/body		1.4 (1:0.9–1.7)	1:1.4*
*Redia*			
Body	L	988 ± 405 (363–2209)	425–1600
	W	190 ± 58 (76–271)	118–450
Pharynx	L	42 ± 13 (24–75)	–
	W	41 ± 15 (21–74)	25–105
Locomotory appendages	L	337 ± 196 (160–908)	138–950
*Metacercaria*			
Whole cyst	D	135 ± 7 (116–145)	132–148
Cystic wall	L	6 ± 3 (3–11)	5

^N^ = natural hosts; ^E^ = experimental host; L, length; W, width; D, diameter; %, proportion; * = estimated from the published drawing.

Measurements are expressed in micrometres.

*Cercaria* (*n* = 36, Fig. 3B–D; Table 2): Body elongate-oval, with maximum width just anterior to ventral sucker. Tegument smooth. Collar well developed, with 37 spines, arranged as in adults. Oral sucker rounded, subterminal. Pharynx ovoid, muscular. Oesophagus long; oesophageal bifurcation just anterior to ventral sucker. Caeca long, with granular content inside, extending up to posterior extremity. Ventral sucker subglobular, muscular, just post-equatorial, slightly larger than the oral sucker; average ratio between suckers width, 1:1.4 (1:1.0–1.6). Penetration glands with granular content, preacetabular, medially distributed, along oesophagus, with canaliculi directed to anterior region, opening in 6 ducts at extremity of oral sucker (Fig. 3D). Paraesophagean glands absent. Cystogeneous glands with rhabditiform content located laterally through body. Genital primordia composed by 2 masses of cells dorsal to ventral sucker. Tail long, larger than body, average ratio between body and tail length 1:1.4 (1:0.9–1.7); ending in a highly contractile finger-like process, slender, presenting tegumental fin-folds. Number and disposition of tail fin-folds not observed. Excretory vesicle small, at posterior extremity of body. Main ducts of excretory system presenting numerous, small calcareous concretions (about 50, 6–9 *μ*m of diameter) between the pharynx and ventral sucker. Details of excretory system, including flame-cell formula, position of excretory pore and caudal excretory duct not characterized.

*Metacercaria* (*n* = 35, Fig. 3E; Table 2): cysts spherical, on average 135 ± 7 *μ*m in diameter; cyst walls composed of 2 layers, the outer layer transparent, about 6 *μ*m thick, and the inner layer opaque, thin, about 2 *μ*m thick.

#### Remarks

The experimentally recovered adult specimens can be readily distinguished from all but 1 species of *Echinostoma* of the ‘*revolutum*’ complex by bearing tiny dorsal collar spines, much smaller than the dorsolateral and lateral ones (see description). Despite the differences in the head collar commented above, the recently described *E*. *pseudorobustum* Valadão *et al*., 2022 presents similar size and body shape. However, this species is differentiated by having a longer forebody (FORE% *c*. 18.9 *vs* 12), oesophagus length as a proportion of body length (OSL% *c*. 10 *vs* 3), head collar as a proportion of body width (CW/BW *c*. 49.5 *vs* 26.1%) and uterine field (OVAR *c*. 41.6 *vs* 28%).

Taking into account the morphological data alone, we initially assigned the new taxon to *E*. *paraensei* because this species is the only one in the ‘revolutum’ complex that also possesses tiny dorsal collar spines (Kostadinova and Gibson, 2000), and also due to similar morphometrics comparing other key morphological features (see Table 1). The geographical origin of the specimens studied (Mesoregion of Belo Horizonte) also led us to preliminarily identify the isolate as *E*. *paraensei*. There are some differences observed between the newly collected specimens and those previously identified as *E. paraensei* by other workers, e.g. body shape (BW%, 19 *vs* 12–13), ratio between the width of oral and ventral suckers (OSW:VSW, 1:2.5 *vs* 1:1.4–2.8) and prepharynx length (PL) (PL: 41 *vs* 72–120 *μ*m in the original description). These differences are attributed here to the evaluation of specimens with different ages (range of 12–109 dpi) in each study as individuals continue to grow after reaching maturity and the use of different techniques for fixing the material (flattening or not of the specimens).

While morphological features suggested the conspecificity of *E*. *maldonadoi* n. sp. and *E*. *paraensei*, molecular data, specifically based on the mitochondrial *nad*1 gene, revealed interspecific levels of nucleotide divergence between the 2 species (see below).

## Molecular study

**Fig. 4.**
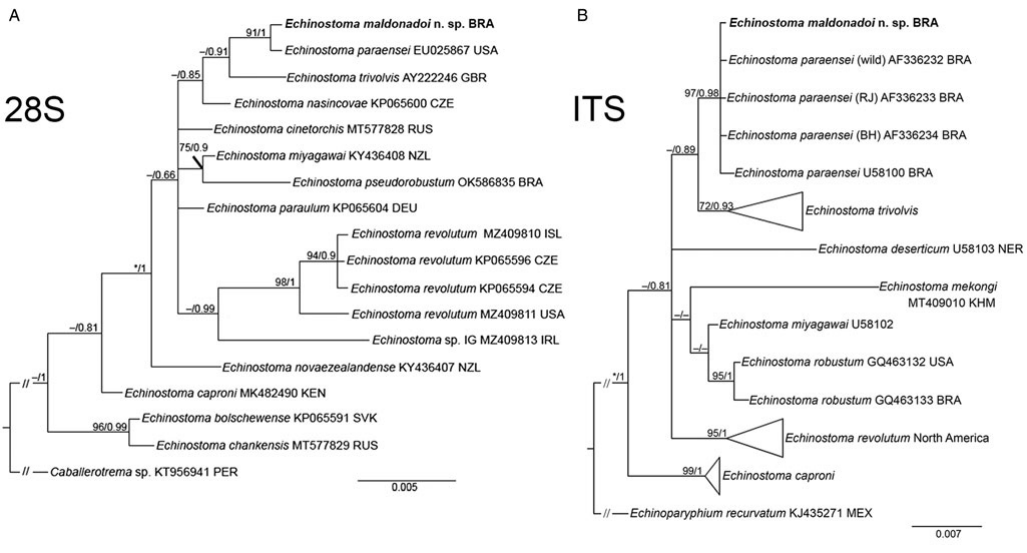
Phylogenetic relationship between *Echinostoma maldonadoi* n. sp. (in bold) and other 37-collar-spined *Echinostoma* species inferred from sequences of 28S rDNA (1051 bp; nucleotide substitution model: HKY + G) (A) and of internal transcribed spacer (ITS) (1001 bp; nucleotide substitution model: K2 + G) (B) based on maximum likelihood (ML) and Bayesian inference (BI) analyses. Nodal support is indicated as ML/BI; values <0.70 (BI) and <70 (ML) are indicated by a dash.

Partial sequences of the 28S rRNA gene (1197 bp), ITS1-5.8S-ITS-2 region (1159 bp), *cox*1 gene (415 bp) and *nad*1 gene (478 bp) were successfully generated for the isolate of 37-collar-spined *E. maldonadoi* n. sp. Phylogenetic trees were reconstructed using BI and ML criteria, but only those obtained through the first approach are shown, including values of nodal support for both analyses. The trimmed 28S alignment was 1051 bp long and included 18 sequences for 14 species of the ‘*revolutum*’ group of *Echinostoma*. The phylogenetic reconstruction based on this gene (Fig. 4A) revealed that *E. maldonadoi* n. sp. clustered in a well-supported clade with an isolate of *E*. *paraensei* maintained under laboratory conditions in the USA, without nucleotide differences between the sequences. The interspecific variation in relation to other *Echinostoma* spp. included in the analyses was 0.3–1.1%.

A dataset containing 9 species of *Echinostoma* was included in the analysis of the nuclear region ITS1-5.8S-ITS2 (trimmed alignment: 1001 bp). *Echinostoma maldonadoi* n. sp. grouped with 3 Brazilian isolates of *E. paraensei* in a strongly supported clade (97/0.98) (Fig. 4B), but the actual phylogenetic affinities with these isolates remained unclear. Moreover, the molecular divergence in relation to the 4 isolates of *E. paraensei* from *N*. *squamipes* was low (0–0.1%). Representatives of this clade appeared as sister to isolates of *E*. *trivolvis*, but the support was low. The interspecific genetic variation in relation to other *Echinostoma* spp. included in the analyses was 0.7–2.6%.

**Fig. 5.**
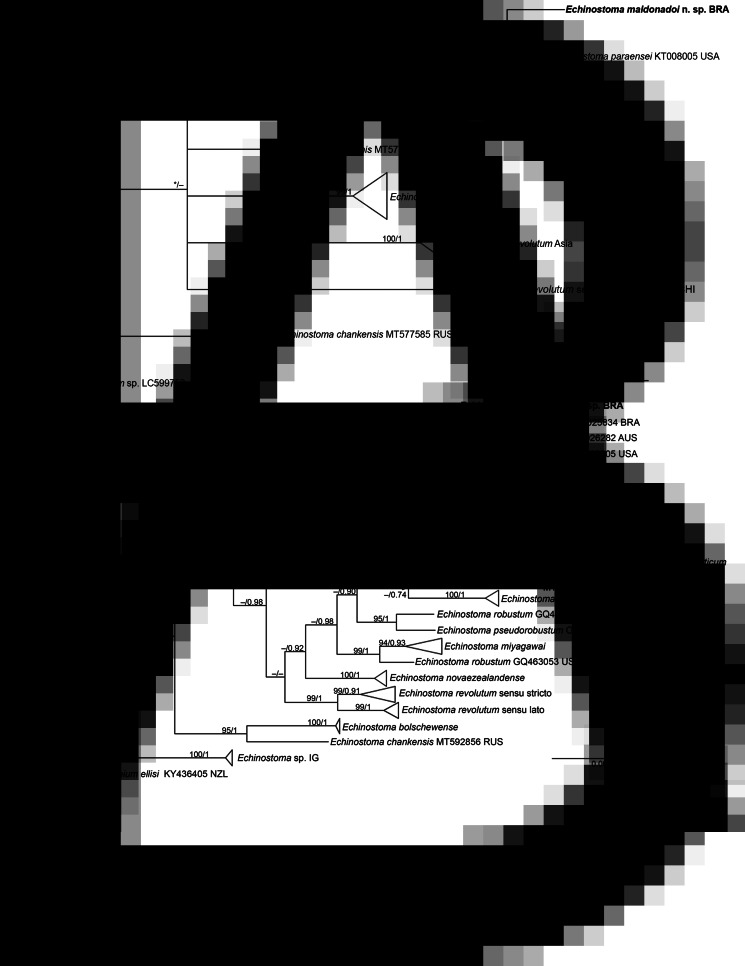
Phylogenetic relationship between *Echinostoma maldonadoi* n. sp. (in bold) and other 37-collar-spined *Echinostoma* species inferred from sequences of *cox*1 mtDNA (366 bp; nucleotide substitution model: HKY + I) (A) and of *nad*1 (438 bp; nucleotide substitution model: HKY + G + I) (B) based on maximum likelihood (ML) and Bayesian inference (BI) analyses. Nodal support is indicated as ML/BI; values <0.70 (BI) and <70 (ML) are indicated by a dash.

The molecular data obtained for the faster evolving mitochondrial genes (*nad*1 and *cox*1) revealed nucleotide differences between the new species and *E. paraensei*. For *cox*1 gene, a trimmed alignment (366 bp) containing 8 species of *Echinostoma* was analysed. The resolution of the tree was poor resulting in a polytomy, which hampered our understanding on the phylogenetic relationships of most taxa (Fig. 5A). Nevertheless, *E*. *maldonadoi* n. sp. was recovered in a strongly supported clade together with *E*. *paraensei*. Moreover, in the *cox*1 tree, *E. maldonadoi* n. sp. + *E. paraensei* is a sister group to *E. caproni*, and this nesting was well-supported by BI (0.96). This clade forms a polytomy with *E. cinetorchis*, *E. miyagawai* and 2 ‘*E. revolutum*’ isolates. The genetic divergence between *E. maldonadoi* n. sp. and the laboratory isolate of *E. paraensei* from the USA was 3.8% (difference at 14 nucleotide positions), which might suggest that they do not belong to the same species. The molecular divergence in relation to other *Echinostoma* spp. ranged from 10.9 to 15.9%.

The molecular analysis based on the *nad*1 gene unequivocally confirmed the distinct species status of *E. maldonadoi* n. sp. The trimmed alignment (438 bp) included 153 sequences of 19 species/species-level lineages of *Echinostoma*. In the well-resolved phylogenetic tree (Fig. 5B), the new species grouped in a strongly supported clade with 3 isolates of *E*. *paraensei*, and both appeared as sister clade to *E*. *nasincovae* (albeit with no support in the ML analysis). The genetic divergence in relation to the isolate of *E*. *paraensei* maintained under laboratory conditions in the USA, and in relation to another isolate of the same species from Brazil, reported by Morgan and Blair (1998*b*), was 6.2% (for 2 isolates) and 6.4% (27 and 28 nucleotides), respectively, which fell into the interspecific range of variability for *Echinostoma* spp. of the ‘*revolutum*’ group. Moreover, the newly described species exhibited a genetic divergence of 12.3–21.0% compared with the other species of the ‘*revolutum*’ group. *Echinostoma maldonadoi* n. sp. can be diagnosed based on unique sites/nucleotides in specific positions of the *nad*1 alignment comparing to isolates of *E*. *paraensei*: 11–A, 20–C, 36–A, 37–T, 47–A, 68–G, 71–C, 81–T, 107–C, 119–A, 122–T, 144–A, 188–G, 197–T, 245–A, 260–T, 275–A, 314–T, 321–A, 337–C, 351–C, 365–T, 372–G, 398–G, 402–C, 405–G, 410–T (see Supplementary file – Alignment of sequences *nad*1 from these isolates).

## Discussion

Our understanding of the species diversity of the genus *Echinostoma* has been improved by integrating molecular and morphological approaches to better delimit species boundaries and consequently address more accurately questions concerning host use, geographical distribution and life cycle (Georgieva *et al*., 2013, 2014; Faltýnková *et al*., 2015; Izrailskaia *et al*., 2021; Valadão *et al*., 2022). A number of studies stressed the presence of several cryptic lineages within the ‘*revolutum*’ species complex of *Echinostoma*, mostly from the Holarctic realm (Detwiler *et al*., 2010, 2012; Georgieva *et al*., 2014; Izrailskaia *et al*., 2021), which presented challenges either for identification of known taxa or the proposal of new taxa with no morphological differential trait to report. Even though cryptic diversity is more common among trematodes than other groups of parasitic helminths (Pérez-Ponce de León and Poulin, 2018), some of the cryptic evolutionary independent lineages found in previous studies of *Echinostoma* spp. may be found morphologically identifiable, once all developmental stages (primarily adults) are described. In the present study, the integration of morphological, molecular and life cycle data made it possible to propose a new species of *Echinostoma*, *E*. *maldonadoi* n. sp., cryptically related to *E*. *paraensei* considering all developmental stages, yet some morphometric differences were found between isolates of the new species and *E*. *paraensei* (see remarks).

From the biological point of view, the intermediate host use is not considered a significant difference between *E. paraensei* and *E. maldonadoi* n. sp. Although natural infection for the former was reported in planorbid snails, *B. grabrata* and *Glyptophysa* sp., in Brazil and Australia, respectively (Lie and Basch, 1967; Morgan and Blair, 1998*a*), the experimental susceptibility of physid snails to *E. paraensei* was reported to the isolate from Belo Horizonte (original description) (Lie and Basch, 1967) and for the isolate from Sumidouro, state of Rio de Janeiro, Brazil (Maldonado *et al*., 2001*b*). Nevertheless, echinostome cercariae were not found among 767 specimens of *Biomphalaria* spp. (including *B. glabrata*) collected in Alvorada de Minas (Coelho *et al*., 2021), the type locality of *E. maldonadoi* n. sp., which may suggest that *E*. *paraensei* and *E*. *maldonadoi* n. sp. are primarily found in planorbid and physid snails, respectively, at least in natural conditions.

A question that emerges from this study, but that is impossible to be answered at the current stage of the knowledge, is whether the subsequent reports of *E. paraensei-*like specimens really correspond to this taxon or *E. maldonadoi* n. sp., or even to another new cryptic species of the genus. Interestingly, some relevant differences between isolates identified as *E. paraensei* were reported regarding: the time of miracidial development, worm burden, number of uterine eggs and parasite distribution in the site of infection (Maldonado *et al*., 2001*a*, 2001*b*, 2005). The possibility that such differences may represent interspecific variation instead of just evident phenotypic plasticity, as previously thought (Maldonado *et al*., 2005), cannot be ruled out. In this sense, future *nad*1 sequencing of this laboratory-maintained isolate of *E. paraensei* is here encouraged for taxonomic validation of the parasite species used in the different experimental studies previously published (Ferraz *et al*., 2012; Gonçalves *et al*., 2013; Monte *et al*., 2016, 2018, 2019; Souza *et al*., 2017*b*).

The utility of the most densely sampled nuclear ribosomal genetic regions, 28S and ITS, and mitochondrial protein-coding genes, *cox1* and *nad*1, were evaluated in molecular phylogenetic studies (Morgan and Blair, 1998*a*, 1998*b*; Georgieva *et al*., 2014; Izrailskaia *et al*., 2021). While all of them agree that mitochondrial genes should be the first choice for barcoding species of *Echinostoma* in the ‘*revolutum*’ group, as nuclear ribosomal genes may not provide enough phylogenetic signal to differentiate closely related species, they differ concerning which mitochondrial marker is more informative. Most of the scientific contributions provided compelling evidence that *nad*1 shows a sufficient gap between levels of intraspecific and interspecific genetic divergence, the so-called barcode gap, allowing the identification of species boundaries (Morgan and Blair, 1998*b*; Georgieva *et al*., 2014, 2017). For this reason, most taxonomic surveys on *Echinostoma* provided *nad*1 sequences (Georgieva *et al*., 2014, 2017; Faltýnková *et al*., 2015; Pantoja *et al*., 2021). However, Izrailskaia *et al*. (2021) argued that *cox*1 may be more informative than *nad*1 and might solve more confidently taxonomic problems within the Echinostomatidae.

In the present study, we reinforce the limitations of ribosomal nuclear DNA for the distinction of closely related species (the new taxon could not be recognized using this dataset) and found only moderate nucleotide divergence (3.83%) using *cox*1 between the sister taxa *E*. *maldonadoi* n. sp. and *E*. *paraensei*, much lower than the 6.3–13.7% and 7.3–16.8% interspecific values reported by Morgan and Blair (1998*b*) and Izrailskaia *et al*. (2021), respectively. These different values may just be biased by the paucity of *cox*1 sequences for *Echinostoma* spp., leaving just a few sequences of closely related sister taxa to be compared. Contrary to that, the divergence values (6.2–6.4%) of the *nad*1 marker between the new species and *E*. *paraensei* were within the interspecific range of variability for *Echinostoma* spp. of the ‘*revolutum*’ group. For instance, in the most comprehensive study involving molecular taxonomy and species delineation in *Echinostoma* spp., Georgieva *et al*. (2014) recognized 17 species/species-level lineages using *nad*1 dataset and found 4.2–21.5% of interspecific divergence. Moreover, identification was achieved for all monophyletic lineages at 3% divergence threshold. These same authors found a mean intraspecific divergence of 0.2–1.8%. More recently, Pantoja *et al*. (2021) reported 0–1.6% of intraspecific divergence for *Echinostoma revolutum s.str.* from Europe and 0.5–1.2% for isolates identified as *Echinostoma* sp. IG. Thus, the divergence found between *E. maldonadoi* n. sp. and *E. paraensei* is more than double from the higher intraspecific divergence previously reported for species of *Echinostoma*. It is also worth noting, that the nucleotide divergence using *nad*1 between *E*. *maldonadoi* n. sp. and the remaining species of the ‘*revolutum*’ group (12.3–21.0%) was considerably higher than compared with its sister taxon (*E*. *paraensei*), which may indicate a very recent speciation event and segregation into these 2 morphologically indistinguishable species.

Our findings support the conclusions of Morgan and Blair (1998*b*) and Georgieva *et al*. (2014) that the best mitochondrial marker for the investigation of phylogenetic interrelationships and identification of cryptic diversity within the ‘*revolutum*’ group of *Echinostoma* is *nad*1. In fact, most internal or terminal clades are strongly supported in our phylogenetic reconstruction. Moreover, the availability of *nad*1 datasets with larger taxonomic and geographical coverage in relation to the remaining markers allows the comparison of genetic distances between 2 putative species with values available for ‘benchmark’ congeners. Nevertheless, finding a ‘genetic yardstick’ remains challenging in trematodes (Nadler and Pérez-Ponce de León, 2011) and even in *Echinostoma*.

Even though the reciprocal monophyly of *E*. *maldonadoi* n. sp. and *E*. *paraensei* could not be assessed as only a single haplotype of the new taxon was herein detected, the knowledge leveraged for the molecular taxonomy of this genus in the last 2 decades, including a clear barcode gap using *nad*1 dataset, suffices for our new species proposal. This hypothesis though may be strengthened by molecular characterization of new isolates and widening the number of target molecular markers, such as encompassing the entire mitochondrial genome.

## Supplementary material

For supplementary material accompanying this paper visit https://doi.org/10.1017/S003118202300001X. click here to view supplementary material
